# Supplementary material for: Modelling the associations between faculty support and academic satisfaction among nursing students: Mediating roles of sleep problems and smartphone addiction
Source: PLOS Ment Health. 2026 Mar 26;3(3):e0000504. doi: 10.1371/journal.pmen.0000504 (PMC13020805; doi:10.1371/journal.pmen.0000504)
Supplement: S1 Text — (DOCX) [file pmen.0000504.s001.docx]

Supplementary file

|  | **Heterotrait-monotrait ratio (HTMT)** |
| --- | --- |
| **Satisfaction with academics <-> Faculty support** | 0.813 |
| **Sleep problem <-> Faculty support** | 0.777 |
| **Sleep problem <-> Satisfaction with academics** | 0.762 |
| **Smartphone addiction <-> Faculty support** | 0.889 |
| **Smartphone addiction <-> Satisfaction with academics** | 0.841 |
| **Smartphone addiction <-> Sleep problem** | 0.903 |

| F& L criterion | **Faculty support** | **Satisfaction with academics** | **Sleep problem** | **Smartphone addiction** |
| --- | --- | --- | --- | --- |
| **Faculty support** | 0.814 |  |  |  |
| **Satisfaction with academics** | 0.740 | 0.808 |  |  |
| **Sleep problem** | -0.727 | -0.686 | 0.837 |  |
| **Smartphone addiction** | -0.803 | -0.758 | 0.827 | 0.839 |
