# Supplementary material for: Modelling the associations between faculty support and academic satisfaction among nursing students: Mediating roles of sleep problems and smartphone addiction
Source: PLOS Ment Health. 2026 Mar 26;3(3):e0000504. doi: 10.1371/journal.pmen.0000504 (PMC13020805; doi:10.1371/journal.pmen.0000504)
Supplement: Attachment — Submitted filename: Point-by-Point_response MODELLING.pdf.pdf [file pmen.0000504.s003.pdf]

RESPONSE TO REVIEWERS' COMMENTS

REVIEWER	COMMENT	RESPONSE	PAGE NUMBER
Reviewer #1:	Title: Modelling the associations between faculty support and academic satisfaction among nursing students: mediating roles of sleep problems and smartphone addiction.<br>This study is interesting; the topic is doable and relevant, and also it is a study that incorporates the time factor. However, there are many points that need more clarifications; the manuscript is also too long and contains formatting inconsistencies, repetitions, and some conceptual ambiguities. Revision is needed.	Authors are grateful for the compliment.<br>Thank you	
	In the introduction, "this study will generate context-specific insights that can inform the development of more supportive educational environments." Have you generated the context -specific insight yet?	Comments addressed as suggested<br>Thank you	4
	Is the smartphone addiction scale is for adolescents? Please justify its use for nursing students (adults).	Although normed on adolescents, the core behavioral and psychological constructs of smartphone addiction such as preoccupation, withdrawal, loss of control, and functional impairment are not age-specific and have been demonstrated to be valid in adult populations.	9
	**Statistical model concerns** You mentioned the p-value in the abstract and inside the paper; unfortunately, I did not have the opportunity to see it. Please attach it in the manuscript or in the supplementary.	All Figures were uploaded differently during submission.<br>Thank you for this comment. All p-values were estimated using bootstrapping (5,000 resamples) in SmartPLS	

		and are now explicitly reported in the Results section (Hypotheses Testing and Mediation Results). The complete bootstrapping output, including path coefficients, t-values, p-values, and confidence intervals, has also been added to the upload differently.	
	An unclear situation has happened between sleep problems and smartphone addiction (positive sign) versus the previous justification. Explain it logically.	We clarify that this result is conceptually and empirically consistent with our hypotheses and prior literature. The positive path indicates that greater sleep problems are associated with higher smartphone addiction, reflecting a risk-reinforcing relationship rather than a beneficial effect. This aligns with evidence that sleep disturbances and excessive smartphone use co-occur and exacerbate one another.	Check Figure 2.
	Reflective measurement model indicators were dropped solely based on loading <0.70; this may alter scale validity. Please report which items were deleted and assess content validity impact.	Deleted items were reported. check page 12-13<br>In reflective measurement models, indicator removal based on low outer loadings <0.70 is a model-refinement decision, not a validity shortcut. An outer loading reflects the proportion of variance in an indicator explained by the latent construct;	12-13

		loadings below 0.70 indicate that more than 50% of the indicator variance is error, which weakens construct reliability and convergent validity. In PLS-SEM, retaining such indicators can inflate measurement error and attenuate structural path estimates. However, psychometric best practice emphasises that statistical criteria must be balanced with theoretical and content considerations. Indicator removal is justified only when:<br>(i) the item contributes weakly to the construct,<br>(ii) its removal improves composite reliability and AVE, and<br>(iii) the remaining indicators still adequately represent the construct's conceptual domain. When these conditions are met, content validity is not compromised but rather preserved through a more internally coherent representation of the latent variable.	
	The interpretation of mediation is right, though the sign on the smartphone path (positive r = 0.177) negates the previous negative impact. Please verify.	Thank you for this observation. The positive sign of the indirect effect (Faculty support → Smartphone addiction → Satisfaction with academics; r = 0.177) does not negate the negative impact of smartphone addiction on academic satisfaction. Rather, it	

		reflects the product of two negative paths: faculty support is negatively associated with smartphone addiction, and smartphone addiction is negatively associated with academic satisfaction. In mediation analysis, multiplying two negative coefficients yields a positive indirect effect, indicating a protective (beneficial) indirect influence of faculty support via reduced smartphone addiction. This interpretation is statistically correct and consistent with the hypothesised model and mediation logic.	
	Discussion is similar to the introduction in many ways. The claims of causality are not permitted, as the design is cross-sectional.	Thank you for your attention to details. Any causal claim made has been corrected.. words such as linked etc have been changed to reflect associations.	
	Please do not exaggerate the role of faculty support as an alternative to institutional support without empirical support.	We thank the reviewer for this important clarification. We have revised the manuscript to remove any implication that faculty support substitutes for institutional support systems. The interpretation has been refined to present faculty support as one interpersonal and contextual factor associated with students' academic experiences, rather than as an alternative to formal	Check throughout the discussion

		institutional structures. All statements implying substitution or compensation have been softened to ensure alignment with the empirical scope of the study and to avoid overinterpretation beyond the data.	
	The data availability statement is a contradiction: it provides a statement that data would be available when requested, but also it has an OSF link. Please make this consistent.	This has been addressed.	20
	**Minor comments** Some references are outdated; please use the current reference as much as possible	We appreciate this comment. While some references may not be recent, they were intentionally retained because they represent seminal, theoretical, or methodological works that remain foundational to the constructs examined and continue to be widely cited in contemporary research. Where appropriate, these sources were complemented with recent empirical studies to ensure the manuscript reflects current scholarship.	
	Explain the abbreviations on first use (e.g., SEM, PLS-SEM)<br><br>Write the correct spellings for words like: "authords" "revelaed", "stadard", "perforamnce",.<br>Thank you so much	This is a standard academic writing requirement which was duly adhered to in the manuscript<br><br>Comments addressed as indicated	11<br><br>5, 6, 11, 14

Reviewer #2:	**General comments:**<br>This is a worthwhile study, especially for resource-limited countries such as Ghana. As it is now written, it is too verbose throughout. Eight hypotheses are not needed to test the important relationships. The important findings are in Table 3.	We thank the reviewer for this constructive comment. As shown in Figure 1 (Conceptual/Structural Model), each hypothesis corresponds to a distinct theoretically specified path within the model, including direct and mediating relationships. The hypotheses were therefore necessary to ensure conceptual clarity, proper model specification, and transparent hypothesis testing in line with SEM conventions. While Table 3 summarises the key statistical results, the hypotheses framework reflects the underlying theoretical structure rather than redundancy.	
	There are spelling and grammatical errors throughout.	The manuscript has been proofread to ensure clarity	
	Specific comments follow:<br>Introduction: The introduction is too long and repetitive.	Authors acknowledge there is lengthy introduction. This is true because the literature review formed part of the introduction. Authors did this to have a well synthesised literature for the manuscript.	
	Eight hypotheses are not needed for this study – one is sufficient, as stated at the top of p. 5: "…the hypothesis that student nurses who perceive strong faculty support are more likely to experience better academic satisfaction, both directly and indirectly, by reducing stress-induced	We appreciate the reviewer's perspective and agree that the overarching hypothesis captures the central premise of the study. However, the eight hypotheses were specified to operationalise this	

	behaviours like excessive smartphone use and promoting healthier routines like improved sleep hygiene." The statistical analysis will parse the relationships.	overarching hypothesis into testable components, corresponding to the individual direct and indirect paths shown in the conceptual model. In structural equation modelling, explicitly stating these component hypotheses is standard practice, as it clarifies model specification, guides mediation testing, and facilitates transparent interpretation of the parsed relationships produced by the statistical analysis. We therefore retained the hypotheses structure to preserve conceptual clarity while acknowledging the unifying overarching hypothesis articulated in the manuscript.	
	P. 3, last paragraph, line 5: Need a sentence break between higher and studies.	Comments addressed accordingly	3
	P. 4, second paragraph: "Few studies have integrated these constructs into a comprehensive model that includes institutional support structures, such as faculty involvement, as potential protective factors." This is an important feature of the present study. Emphasize this in a more succinct introduction.	Comments addressed	3
	P.5, last two lines are not a complete sentence.	Well noted and addressed	
	P.6, line 1: reinforces (singular).	Concern addressed accordingly	5
	P.6, paragraph 2, line 4: performance is misspelled.	Concern addressed accordingly	5

	P.6, paragraph 2, last line: author is misspelled.	Concern addressed accordingly	5
	P.6, Hypotheses 1-3 can be combined into a single hypothesis: "Faculty support is positively associated with satisfaction with academics and negatively associated with sleep problems and smartphone addiction." The subsidiary hypotheses 4-8 are redundant and contribute little to the overall issue of the importance of faculty support to nursing students' well-being. As well, their accompanying text can be condensed considerably in the description of the single hypothesis, as suggested above.	Authors think otherwise and want to work with the structural paths presented in Figure 1.	
	Methods: P. 10, paragraph 1, last sentence regarding students' demographics is redundant; this already has been stated in the Introduction.	Thank you for the comment. Concerns addressed accordingly	9
	Analytical Procedures: Overall, the reporting is very statistically dense and will be difficult for the average reader of PLOS Mental Health to comprehend. Can this section be simplified?	We appreciate the reviewer's concern regarding readability. The Analytical Procedures section was intentionally reported in accordance with established PLS-SEM guidelines to ensure methodological transparency, rigour, and reproducibility.	
	P. 11, paragraph 1, first sentence: "…missing values were assessed and addressed appropriately…" How did you do this? Without the procedure, I can't judge if they were done appropriately.	This was stated	11-12
	P. 12, paragraph 1, line 10: Delete "the."	Action taken accordingly	12
	P. 12, paragraph 2, line 4: "…multicollinearity issues was not a in the path model…" Was should be were.	Comments addressed accordingly	12

	Also, word missing after “not a” – not a what?		
	P. 13, paragraph 2, line 2: Need space between procedure and with.	Comments addressed accordingly	13
	P. 13, paragraph 3, line 1: Paths misspelled.	Comments addressed accordingly	13
	Results: These need to be re-written after the number of hypotheses is reduced to the single, important one.	Authors maintained hypotheses based on the structural model.	
	Discussion: Discussion should be singular (not Discussions).	Comments addressed accordingly	15
	The discussion is of reasonable length, but it should be revised in accordance with the revised	Thank you. Authors have mode some corrections based on previous comments.	
	Results section. Limitation in this review: I suggest renaming this to “Strengths and Limitations” and placing it before the section,	This has been addressed	18
	“Implications for Nursing Education and Research.” There are strengths to this report that should be highlighted, as well as limitations. Some of the strengths have been noted in the above comments. Re-emphasize these.	The strength and limitation have been highlighted but limited to only the method used.	18
	Conclusion: Conclusions (should be plural).	Comments addressed accordingly	17
	Line 5: Should be “…mediators, illustrating two of the behavioural…” There are likely others not examined.	Thank you. Authors examined only those whose hypotheses were stated and literature reviewed for.	
	Declarations: These are abbreviations rather than declarations.	Abbreviation have been correctly stated to reflect reviewer’s concern.	19

	Author contribution: contributions (should be plural). I suggest adding at the end of this paragraph a statement about any use of artificial intelligence in the literature review and/or the writing of the article.	Comments addressed accordingly	19
	References: Please format the references consistently, in journal format. Some doi's seem excessively long; please check them, e.g., ref's 2 & 4.	This has been addressed	21-25
	Figures and Tables: Figure 1 (structural model) is useful. The main elements of Figure 2 (e.g., path coefficients) can be incorporated into Figure 1, and Figure 2 can be eliminated.	Authors wish to maintain both. The two are serving as two different purpose and we wish to maintain them.	
	Table 1 can be simplified in accordance with the reduction of 8 hypotheses to one overarching hypothesis, as described above.	Authors wish to maintain the hypotheses based on our structural model..	
	Table 2 should be Table 1 and its discussion re-ordered in the text. (An interesting, later analysis might be to examine whether any of the demographic variables moderated the findings; e.g., did male students have a different pattern than females? Did college level moderate the findings? Age, marital status, and religion do not have sufficiently broad distributions to consider them as possible moderating variables.)	Authors confirm that the place for Table 1 and 2 are correct.<br><br>Thank you for this suggestion. Future studies perhaps may address these moderating roles. Thank you.	
	Table 3 is useful as a complement to Figure 1. Table 4 has different (hypothesized) path coefficients than Table 3 for the same relationships, so I'm not clear on which is more important. When the overall analysis is simplified according to a single, overarching hypothesis, Tables 3 and 4 can be revised.	We thank the reviewer for this observation. Tables 3 and 4 serve distinct analytical purposes within the model. Table 3 presents the direct structural path coefficients for the hypothesised relationships, while Table 4 reports the indirect (mediation) effects, which are necessarily estimated separately and therefore	

		differ in magnitude and sign from the direct effects. These differences do not indicate inconsistency but reflect standard mediation analysis in SEM. Collapsing the tables into a single overarching hypothesis would reduce clarity regarding the specific direct and indirect pathways specified in the conceptual model. We therefore retained the current structure to preserve analytical transparency.	
