# Supplementary material for: Modelling the associations between faculty support and academic satisfaction among nursing students: Mediating roles of sleep problems and smartphone addiction
Source: PLOS Ment Health. 2026 Mar 26;3(3):e0000504. doi: 10.1371/journal.pmen.0000504 (PMC13020805; doi:10.1371/journal.pmen.0000504)
Supplement: Attachment — Submitted filename: Point-by-Point_response MODELLING_09_03.pdf [file pmen.0000504.s007.pdf]

RESPONSE TO REVIEWERS' COMMENTS

REVIEWER	COMMENT	RESPONSE	PAGE NUMBER
	1) Make the heading "Discussions" singular.	Authors are grateful for the compliment and further suggestions<br>Thank you<br>Suggestions taken and acted on accordingly	Page 17
	2) Change "Declarations" to "Abbreviations."	Authors have changed declaration to abbreviations as suggested	Page 20
	3) Add your statement about the use of A.I.	A statement on the use of AI has been included in the manuscripts as indicated	Page 20
