# Supplementary material for: Modelling the associations between faculty support and academic satisfaction among nursing students: Mediating roles of sleep problems and smartphone addiction
Source: PLOS Ment Health. 2026 Mar 26;3(3):e0000504. doi: 10.1371/journal.pmen.0000504 (PMC13020805; doi:10.1371/journal.pmen.0000504)
Supplement: Attachment — Submitted filename: Point-by-Point_response MODELLING.docx [file pmen.0000504.s004.docx]

RESPONSE TO REVIEWERS’ COMMENTS

| REVIEWER | COMMENT | RESPONSE | PAGE NUMBER |
| --- | --- | --- | --- |
| #2 | I commented on many details that were discrepant, including multiple spelling errors, erroneous DOIs in the references, etc. While most have been corrected, there still are some remaining.  For example, the DOI for ref. 29, suspiciously long, does not work, at least for me. Every DOI in the reference list needs to be checked again, because each one must work.  The authors also indicate they provided a statement about the use of AI after the listing of author contributions, but it is missing from the revised manuscript. Please provide a detailed statement regarding whether AI was used at any stage of manuscript preparation, including the author responses to the reviews of the original version. | Authors are grateful for the compliment and further suggestions  Thank you  Authors have carefully checked and corrected every DOI in the reference list. However, reference number 1011,12,13 and 28 do not have DOIs attached to the publications. The revised DOIs have been indicated in tracked changes  Although generative AI tools were not primarily used in the write up, authors can confirm that Grammarly and QuillBot softwares were used for editing and paraphrasing purposes. | Page 22 to 29 |
