# Supplementary material for: Modelling the associations between faculty support and academic satisfaction among nursing students: Mediating roles of sleep problems and smartphone addiction
Source: PLOS Ment Health. 2026 Mar 26;3(3):e0000504. doi: 10.1371/journal.pmen.0000504 (PMC13020805; doi:10.1371/journal.pmen.0000504)
Supplement: Attachment — Submitted filename: RESPONSE TO REVIEWERS_PLOSONE_FEB 11.docx [file pmen.0000504.s005.docx]

**AI and Language Editing Disclosure Statement**

The authors confirm that generative AI tools were not used in the development of the intellectual content of this manuscript or in the generation, analysis or interpretation of the data. Grammarly and Quill Bot were used solely for language editing and paraphrasing to enhance clarity and readability. The authors take full responsibility for the content and integrity of the manuscript.
